# Revisiting CD8 T-cell ‘Memory Inflation’: New Insights with Implications for Cytomegaloviruses as Vaccine Vectors

**DOI:** 10.3390/vaccines8030402

**Published:** 2020-07-22

**Authors:** Rafaela Holtappels, Kirsten Freitag, Angelique Renzaho, Sara Becker, Niels A.W. Lemmermann, Matthias J. Reddehase

**Affiliations:** Institute for Virology and Research Center for Immunotherapy (FZI) at the University Medical Center of the Johannes Gutenberg-University Mainz, 55131 Mainz, Germany; kfreitag@uni-mainz.de (K.F.); renzaho@uni-mainz.de (A.R.); sarbecke@uni-mainz.de (S.B.); lemmermann@uni-mainz.de (N.A.W.L.); matthias.reddehase@uni-mainz.de (M.J.R.)

**Keywords:** avidity maturation, central memory CD8 T cells (TCM), cytomegalovirus, effector memory CD8 T cells (TEM), conventional TEM (cTEM), inflationary TEM (iTEM), KLRG1, memory inflation, vaccine vector

## Abstract

Murine models of cytomegalovirus (CMV) infection have revealed an exceptional kinetics of the immune response. After resolution of productive infection, transient contraction of the viral epitope-specific CD8 T-cell pool was found to be followed by a pool expansion specific for certain viral epitopes during non-productive ‘latent’ infection. This phenomenon, known as ‘memory inflation’ (MI), was found to be based on inflationary KLRG1^+^CD62L^−^ effector-memory T cells (iTEM) that depend on repetitive restimulation. MI gained substantial interest for employing CMV as vaccine vector by replacing MI-driving CMV epitopes with foreign epitopes for generating high numbers of protective memory cells specific for unrelated pathogens. The concept of an MI-driving CMV vector is questioned by human studies disputing MI in humans. A bias towards MI in experimental models may have resulted from systemic infection. We have here studied local murine CMV infection as a route that is more closely matching routine human vaccine application. Notably, KLRG1^−^CD62L^+^ central memory T cells (TCM) and conventional KLRG1^−^CD62L^−^ effector memory T cells (cTEM) were found to expand, associated with ‘avidity maturation’, whereas the pool size of iTEM steadily declined over time. The establishment of high avidity CD8 T-cell central memory encourages one to pursue the concept of CMV vector-based vaccines.

## 1. Introduction

Human cytomegalovirus (hCMV) is the prototype member of the β-subfamily of herpesviruses. In the immunocompetent host, infection is well-controlled by the immune system, despite the expression of viral immune evasion proteins specialized to interfere with essentially all mechanisms of intrinsic antiviral defense as well as of innate and adaptive immunity [1,2,3,4,5]. After clearance of productive infection, the viral genome is maintained in a non-replicative state, referred to as latent infection, or briefly as ‘latency’ [6,7,8,9]. Overt hCMV multiple-organ disease and organ failure resulting from cytopathogenic tissue infection is restricted to congenital or perinatal infection of immunologically immature fetuses and premature infants, respectively, [10,11] as well as to immunocompromised patients. A clinical challenge is hCMV reactivation from latency [12,13,14] under conditions of iatrogenically compromised immunity. This includes recipients of solid organ transplantations (SOT) and hematopoietic cell transplantation (HCT) [15,16,17].

The mouse model using murine cytomegalovirus (mCMV) has already proven its value for defining basic principles of CMV pathogenesis, immune control, and experimental immunotherapy (reviewed in [18]). A prominent feature of the immune response to mCMV is ‘memory inflation’ (MI). In essence, it was found that the acute immune response is followed by a contraction phase, during which numbers of epitope-specific CD8 T cells decline before frequencies of cells specific for certain epitopes increase steadily over time [19,20,21,22,23,24]. The epitope-selectivity of MI, distinguishing ‘MI-driving’ and ‘MI-neutral’ epitopes, is still under investigation. The current hypothesis of episodes of CD8 T-cell restimulation by presentation of antigenic peptides is based on the finding that genes in latent viral genomes are temporarily desilenced in a stochastic off–on–off fashion, independent of the reactivation of productive gene expression (reviewed in [25]). Episodes of desilencing lead to a low-level of ‘transcripts expressed in latency’ (TEL). If a TEL happens to encode an antigenic peptide presented by an MHC class-I (MHC-I) molecule, it can potentially drive MI, provided that antigen processing is efficient, and that the functional avidity of the CD8 T cells is high enough to also detect limited peptide presentation [26,27,28]. All these parameters must fit, and this condition appears to be met only by a few viral epitopes.

MI gained broad attention by proposing CMV(s) as a new class of vaccine vector(s), in which antigenic peptides of target pathogens or tumors replace endogenous MI-driving peptides or are expressed under the control of a viral promoter that is frequently desilenced during viral latency. Proof-of-concept was first provided for foreign epitopes expressed under the control of the mCMV *ie2* enhancer-promoter [29]. Since then, numerous studies demonstrated protective immunity induced by recombinant CMV vectors in experimental settings (reviewed in [30,31]). It is the aim of T cell-based vaccination to generate a large pool of long-lived CD62L^+^ central memory cells (TCM) that have stem cell capacity and high proliferative potential for rapidly mounting a recall response upon vaccine pathogen encounter [32,33]. While the term MI might suggest an expansion of TCM, MI was found to be based on activated KLRG1^+^CD62L^−^ cells that depend on frequent restimulation. These cells were originally characterized as short-lived effector cells (SLEC) [34]. More recently, it was reported that inflationary KLRG1^+^CD62L^−^ cells in latent infection differ from KLRG1^+^CD62L^−^ SLEC of the acute response, in that they have an extended life span due to IL-15-mediated expression of the anti-apoptotic protein Bcl-2, which makes them ‘memory cell-like’ [35]. We propose here to name these cells ‘inflationary effector-memory T cells’ (iTEM), to emphasize their key characteristic that distinguishes them from the conventional KLRG1^−^CD62L^−^ effector-memory T cells (cTEM).

The decisive question for medical translation will be if the concept of MI-driving CMV-vector vaccines works also in humans. All previous experimental work has taken it for granted that MI is a ‘hallmark’ of CMV infections in general. This assumption has been challenged recently in an overview of decades of human studies, ending up with the conclusion “that there is only limited evidence supportive of ‘memory inflation’ occurring in humans” [36]. It thus appears that infection conditions, which drive MI in mouse models, are not consistently met in human infection. If one looks for a common denominator in mouse models in which MI was observed, high-dose systemic infection via the intraperitoneal or intravenous routes as well as systemic virus spread in transiently immunocompromised HCT recipients stand out. These conditions all lead to a high load of latent viral genomes in tissues and a correspondingly high TEL activity that provides antigenic peptides for frequent episodes of CD8 T-cell restimulation favoring the expansion of iTEM. However, the licensing of CMV vector-based vaccines will be unlikely when immunity depends on systemic infection for driving MI.

As a more realistic model, we have here studied intraplantar infection. The mouse planta as an application site at an extremity more closely matches the favored vaccine application site in humans, namely subcutaneous and/or intramuscular injection into the upper arm. Such local infections do not bypass draining lymph nodes, the key checkpoint for virus replication and first lymphoid site of priming an antiviral immune response [37,38,39,40]. Our data show that MI constituted by iTEM does not occur after local infection of immunocompetent mice. Whereas the iTEM pool steadily declined over time, proportions of cTEM, and even more of TCM, rose over time. This population dynamics was accompanied by ‘avidity maturation’ in that CD8 T cells with high functional avidity, capable of recognizing infected tissue cells, expanded preferentially. From this, we conclude that the concept of using CMVs as vaccine vectors is still worth pursuing, even though such vaccines will not work by iTEM-based MI, as proposed previously, but rather by establishing a pool of high avidity TCM.

## 2. Materials and Methods 

### 2.1. Mice, Viruses and Infection Procedures

Female BALB/cJ mice were bred and housed at the translational animal research center (TARC) of the University Medical Center of the Johannes Gutenberg-University Mainz under specified-pathogen-free (SPF) conditions. Animal experiments were approved by the ethics committee of the ‘Landesuntersuchungsamt Rheinland-Pfalz’ according to German federal law §8 Abs. 1 TierSchG (animal protection law), permission numbers 177-07/G09-1-004 and 177-07/G14-1-015. Mice were used at the age of 8-to-12 weeks. For intraplantar infection, which combines subcutaneous and intramuscular infection, purified virus was injected into the left hind footpad. Both intraplantar and intraperitoneal infections were performed with 10^5^ plaque-forming units (PFU) of mCMV-BAC^W^ (bacterial artificial chromosome-derived virus MW97.01) [41]. For cell culture assays, murine embryonal fibroblasts (MEF) were infected using mCMV-BAC^W^-derived recombinant viruses with deletions of immune evasion genes [42]. For all experiments, BAC sequence-free [41], high titer virus stocks were prepared from infected MEF by standard protocol [43,44].

### 2.2. Experimental Hematopoietic Cell Transplantation

Syngeneic HCT was performed, as described in greater detail previously [43]. Briefly, recipient female BALB/cJ mice were subjected to total-body γ-irradiation with a single dose of 6.5 Gy, and 5 × 10^6^ BALB/cJ donor-derived tibial and femoral bone marrow cells were infused into the tail vein, followed by intraplantar infection with 10^5^ PFU of mCMV.

### 2.3. Quantitation of Infectious Virus

Infectious virus (expressed as PFU) was quantitated for whole organ homogenates by a virus plaque assay performed on monolayers of MEF, making use of increasing the sensitivity by the method of ‘centrifugal enhancement of infectivity’ [43].

### 2.4. Quantitation of Viral Genomes

DNA from latently infected organs was extracted with the DNeasy blood and tissue kit (catalog no. 69504, Qiagen, Hilden, Germany), according to the manufacturer’s instructions. Viral genomes were quantitated in absolute numbers by *M55*-specific and *pthrp*-specific qPCRs normalized to a log_10_-titration of standard plasmid pDrive_gB_PTHrP_Tdy, as described previously [44].

### 2.5. Peptides

Synthetic peptides corresponding to reported epitopes presented by MHC-I molecules K^d^, D^d^, and L^d^ are derived from the mCMV open reading frames (ORFs) m04, m18, M45, M83, M84, M105, m123/IE1, m145, and m164 (listed in [26,27]). Custom peptide synthesis with a purity of >80% was performed by JPT Peptide Technologies (Berlin, Germany). Synthetic peptides were exogenously loaded on P815 mastocytoma cells (*H-2^d^*) or BALB/c (*H-2^d^*) MEF, as specified in the figure legends, for use as stimulator cells in the enzyme-linked immunospot (ELISpot) assay described below.

### 2.6. Preparation of Single-Cell Suspensions from Lungs and Spleen

Mice were lethally anesthetized by carbon dioxide inhalation, and mononuclear leucocytes from lung tissue were isolated essentially as described [43], with modifications. In brief, lungs were perfused via the right ventricle to remove circulating cells from the capillary bed of the lungs. Lungs were excised, tracheae, bronchi, and pulmonary lymph nodes were discarded, and the lung lobes were minced. The digestion of tissue derived from 4–5 lungs was performed in 15 mL of supplemented DMEM, containing collagenase A (1.6 mg/mL; catalog no. 10 103 586 001, Roche, Mannheim, Germany) and DNase I (50 µg/mL; catalog no. DN-25, Sigma-Merck, Darmstadt, Germany) for 1 h at 37 °C with constant stirring. Mononuclear leucocytes were enriched by density gradient centrifugation for 30 min at 760× *g* on Lymphocyte Separation Medium Histopaque-1077 (catalog no. 10771, Sigma-Merck). For preparing single-cell suspensions of splenocytes, spleens were minced and passed through a cell strainer, followed by the lysis of erythrocytes.

### 2.7. ELISpot Assay

Frequencies and avidities of mCMV-specific CD8 T cells were determined by an IFN-γ-based, 18-hr ELISpot assay, as described [38]. In brief, graded numbers of immunomagnetically purified CD8 T cells from spleen and lung tissue were stimulated for IFN-γ secretion in triplicate assay cultures. High- and low-avidity m164-specific cytolytic T-lymphocyte lines (CTLL) were generated by repetitive restimulation with synthetic m164 peptide at molar concentrations of 10^−10^ M and 10^−8^ M, respectively [26], and assayed for IFN-γ secretion accordingly. Stimulator cells were either P815 cells or MEF, exogenously loaded with synthetic peptides at the saturating loading concentration of 10^−6^ M, or with graded peptide concentrations. Effector cell stimulation under the influence of immune evasion proteins was performed with MEF centrifugally infected with 0.2 PFU per cell, which corresponds to a multiplicity of infection (MOI) of 4 [43], of the indicated recombinant viruses. After incubation for 90 min, the infected MEF were used as stimulator cells in the ELISpot assay.

### 2.8. Cytofluorometric Analyses

Single-cell suspensions were prepared from spleen and lung tissue as described above. Unspecific staining was blocked with unconjugated anti-FcγRII/III antibody (anti-CD16/CD32; clone 93, eBioscience, San Diego, CA, USA), and cells were stained with the following antibodies for multi-color cytofluorometric analyses: ECD-conjugated anti-CD8α (clone 53-6.7; Beckman Coulter, Krefeld, Germany), FITC-conjugated anti-KLRG1 (clone 2F1; eBioscience), and PE-Cy7-conjugated anti-CD62L (clone MEL-14; Beckman Coulter). Phenotypic characterization of peptide-specific CD8 T cells was performed using PE-conjugated MHC-I dextramers H-2Ld/YPHFMPTNL (IE1) and H-2Dd/AGPPRYSRI (m164) (Immudex, Copenhagen, Denmark). Cytofluorometric analyses were performed with flow cytometer FC500 and CXP analysis software (Beckman Coulter).

### 2.9. Statistics

For longitudinal immune response analyses, groups of age-matched mice were randomized before treatment, and data for the indicated read-out times after infection represent the experimental average for pooled samples. Frequencies (most probable numbers) of cells responding in the ELISpot assay (see above) and the corresponding 95% confidence intervals were calculated by intercept-free linear regression analysis from the linear portions of regression lines, based on spot counts from triplicate assay cultures for each of the graded cell numbers seeded [38]. Spots were counted automatically based on standardized criteria using ImmunoSpot S4 Pro Analyzer (Cellular Technology Limited, Cleveland, OH, USA). For analyzing the dynamics of epitope-specific CD8 T-cell subpopulations, a trend analysis by linear regression was performed with Graph Pad Prism 6.04 (Graph Pad Software, San Diego, CA, USA). Rising and declining trends are reflected by positive and negative slopes of regression lines, respectively. Trends were considered as statistically significant by non-overlapping 95% confidence intervals and *p*-values of < 0.05, compared to the ‘null hypothesis’ of having no slope.

## 3. Results

### 3.1. Memory CD8 T-cell Population Dynamics Reveals Continuous Loss of iTEM and Increase in cTEM and TCM after Local Infection of the Immunocompetent Host

CMV vectors that induce MI are proposed to represent a new class of vaccines by amplifying memory cells over time. Much work on MI in the mouse model, however, was based on systemic high-dose infection after intraperitoneal or intravenous virus application. Although this is of academic interest in an experimental animal model, a medical translation of the findings is unlikely, because such routes of vaccine application are not practicable in humans. As an experimental model for a CMV vector-based vaccine that does not depend on systemic infection, we infected young adult BALB/c mice with mCMV at one hind-foot planta. Such an intraplantar infection represents a site and route more comparable to subcutaneous and/or intramuscular vaccine administration into the upper arm of human vaccinees.

Draining regional lymph nodes, the popliteal lymph node (PLN) in the case of intraplantar infection, represent the first lymphoid priming site of an antiviral immune response [37,38]. After administration into the planta, mCMV rapidly reaches the PLN via the lymph stream and infects CD169^+^ macrophages in a demarcated zone beneath the subcapsular sinus in an inoculum dose-dependent manner [37,38,39,40]. Intranodal viral gene expression, which is indicative of productive infection and antigen expression, was detectable already on day 1, and with a lower-limit inoculum virus dose of 10 PFU [38]. Viral epitope-specific CD8 T cells in the PLN increased in frequency in a dose-dependent manner from day 3 onward and peaked on day 7, corresponding with control of acute infection. Importantly, both intranodal viral gene expression and CD8 T-cell frequency in the PLN reached a plateau at an inoculum virus dose of 10^4^–10^5^ PFU, so that higher doses do not improve the local immune response [38]. On day 7, CD44^+^CD62L^−^KLRG1^+^ CD8 T cells are present in the draining PLN and in the spleen, but not in non-draining control LN, and were found to protect against mCMV infection of immunocompromised recipient mice upon adoptive cell transfer (reviewed in [27]).

**Figure 1:**
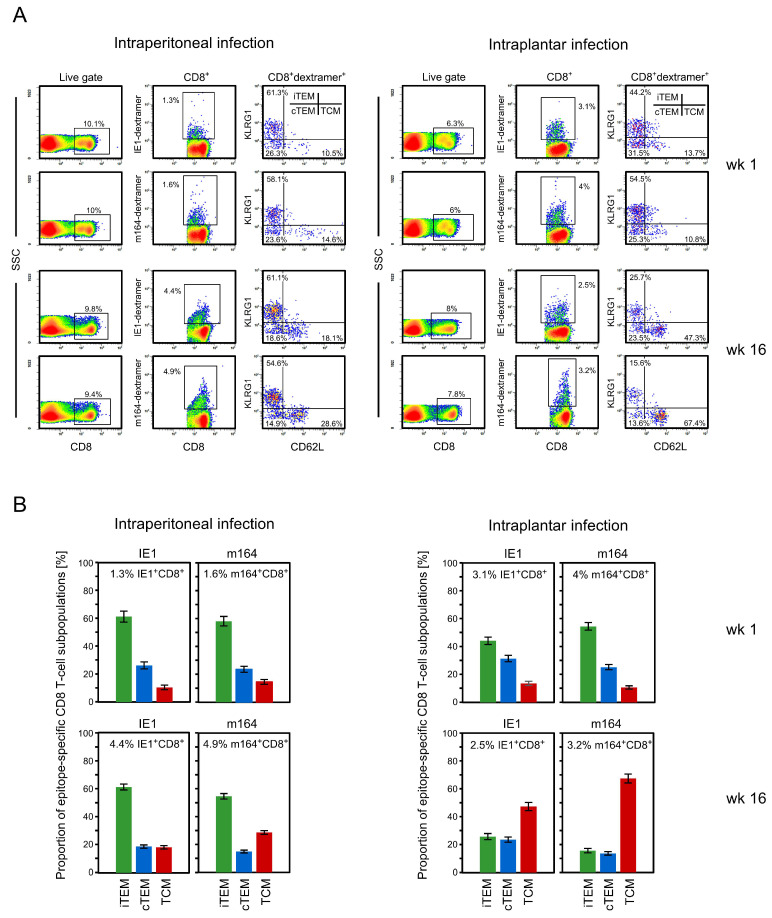
Comparison of intraperitoneal and intraplantar infection. Immunomagnetically-purified CD8 T cells derived from the spleens of immunocompetent BALB/c mice (pool of 4–5 mice per time of analysis) were tested at the indicated times, after intraperitoneal or intraplantar infection with 10^5^ plaque-forming units (PFU) of murine cytomegalovirus (mCMV). (**A**) Cytofluorometric analysis for defining activation phenotypes of IE1 and m164 epitope-specific CD8 T cells. After setting a ‘live gate’ in the forward vs. sideward scatter (SSC) plot (not shown), a second gate was set on CD8 T cells and a third gate was set on CD8 T cells stained with the respective MHC-I-peptide dextramer. Gated cells were tested for the expression of the activation markers CD62L and KLRG1, defining the populations inflationary effector-memory T cells (iTEM) (KLGR1^+^CD62L^−^), conventional effector memory T cells (cTEM) (KLGR1^−^ CD62L^−^), and central memory T cells (TCM) (KLGR1^−^CD62L^+^). (**B**) Proportions of the activation phenotypes. iTEM (green bars), cTEM (blue bars), TCM (red bars). The standard error is indicated.

After the resolution of productive infection, viral latency is established, as documented for spleen and lungs by long-term maintenance of viral genomes in absence of infectious virus (Figure S1). In a first approach, we compared the widely used intraperitoneal infection, serving as a positive ‘reference model’ for MI, with intraplantar infection at an identical infection dose of 10^5^ PFU (Figure 1). It is important to note that the comparison was made in the same experiment with randomized mice and the same virus batch, to avoid any variables other than the route of infection. The CD8 T-cell response to the two MI-driving epitopes IE1 (YPHFMPTNL-L^d^) and m164 (AGPPRYSRI-D^d^) in the *H-2^d^* haplotype [45] was quantitated in the spleen in the acute phase of infection after 1 week, as well as during latent infection after 4 months, by cytofluorometric staining with the respective MHC-peptide multimers, combined with activation markers CD62L and KLRG1 (gating strategy and original data: Figure 1A, summary of results: Figure 1B).

MI specific for these two epitopes was reproduced for intraperitoneal infection, and KLRG1^+^CD62L^−^ iTEM dominated over KLRG1^−^CD62L^−^ cTEM and KLRG1^−^CD62L^+^ TCM. In contrast, after intraplantar infection, proportions of epitope-specific cells in the spleen were higher than after intraperitoneal infection in the acute phase, but MI, as defined by increasing numbers of cells expressing epitope-specific T-cell receptors (TCR), was not observed during latent infection. This finding is in agreement with missing or less pronounced MI noted for intraplantar infection by Snyder and colleagues [46]. An analysis of the CD8 T-cell activation phenotypes revealed a reciprocal image to intraperitoneal infection in that after intraplantar infection TCM dominated over iTEM and cTEM during latent infection. Thus, within an almost stable overall pool of epitope-specific CD8 T cells, the central memory pool was expanded at the expense of the iTEM pool.

**Figure 2:**
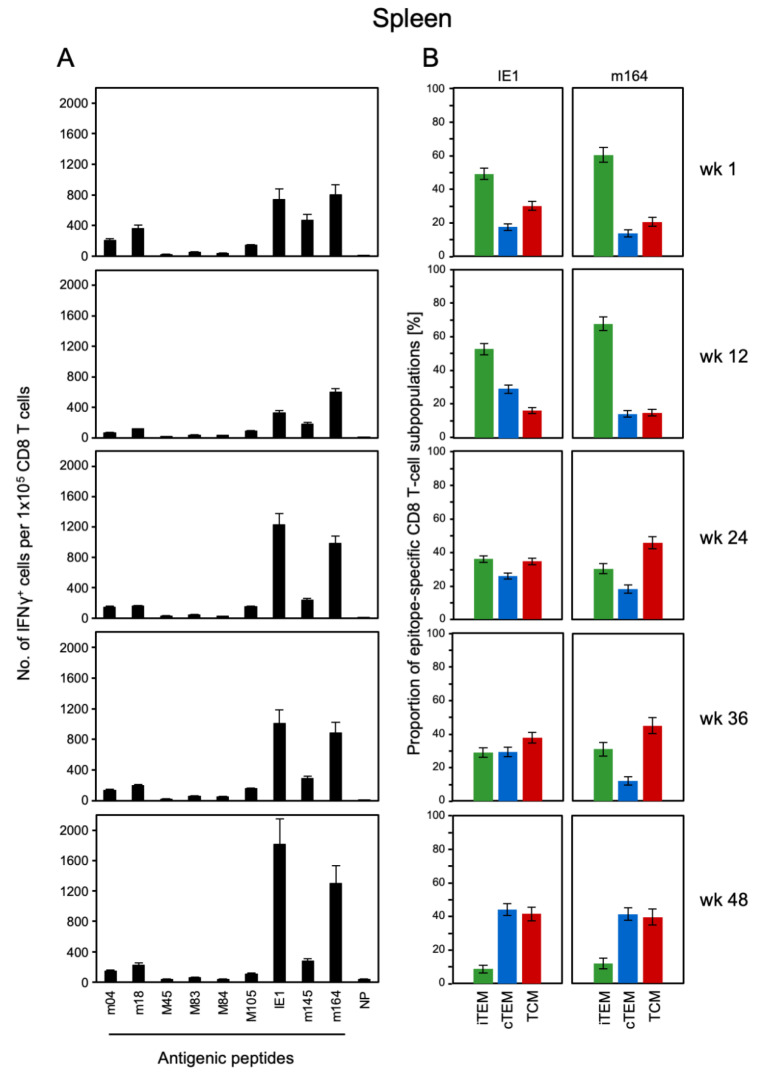
Kinetics of CD8 T-cell specificity repertoires and activation phenotypes in the spleen. At the indicated times after intraplantar infection with 10^5^ PFU of mCMV, spleen-derived CD8 T cells were immunomagnetically purified from pools of 4–5 BALB/c mice. (**A**) Frequencies of mCMV epitope-specific CD8 T cells responding in the ELISpot assay with IFN-γ secretion to stimulation with P815 cells exogenously-loaded with the indicated synthetic peptides at saturating concentration of 10^−6^ M. NP, control with no peptide. Bars represent the most probable numbers, and error bars indicate the 95% confidence intervals determined by linear regression analysis. (**B**) Proportions of activation phenotypes of epitope-specific CD8 T cells defined by cytofluorometric analysis of KLRG1 and CD62L expression within gated dextramer-stained cells (for more detail, see Figure 1). iTEM (green bars), cTEM (blue bars), TCM (red bars). The standard error is indicated.

**Figure 3:**
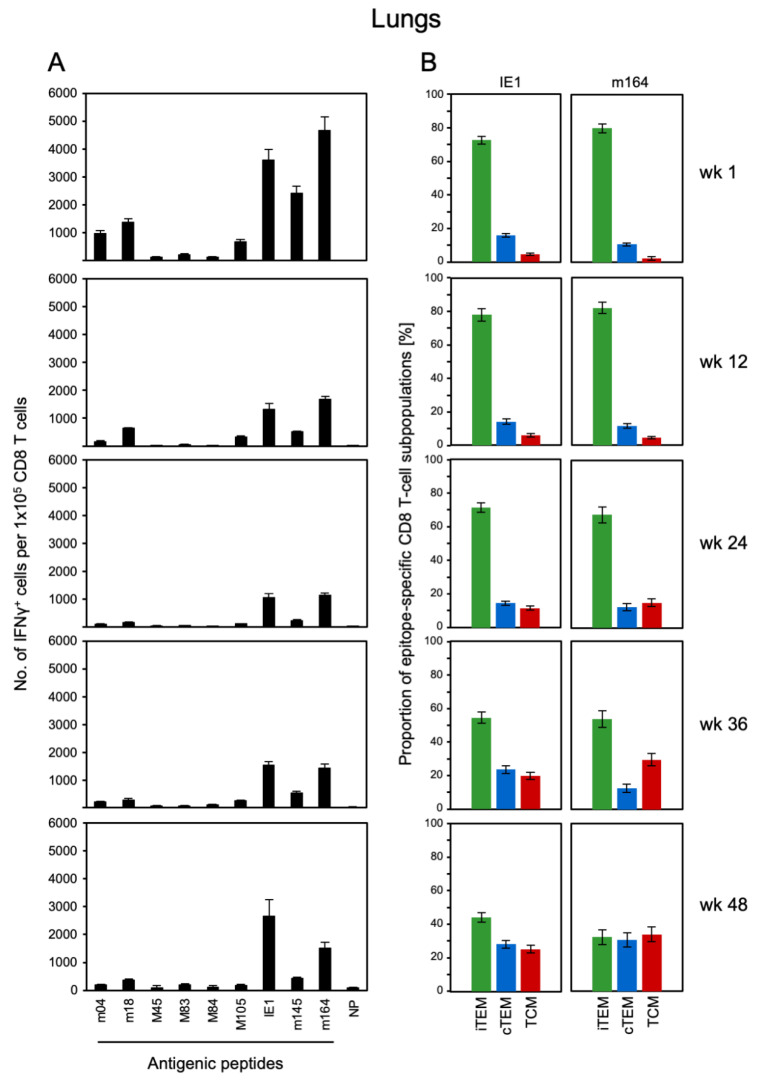
Kinetics of CD8 T-cell specificity repertoires and activation phenotypes in the lungs. Corresponding to the data shown in Figure 2 for the spleen, frequencies of viral epitope-specific functional CD8 T cells (**A**) and proportions of iTEM, cTEM, and TCM (**B**) were determined for pulmonary CD8 T cells from the same pools of mice. For more detail, see Figure 2.

As intraplantar infection is closer to a realistic application of CMV vector-based vaccines, we focused our interest on this infection route and studied the dynamics of the CD8 T-cell response in spleen (Figure 2) and lungs (Figure 3) in the long-term course, monitoring a large cohort of infected mice by sampling at 3-month intervals. Recent work by Smith and colleagues [47] in a model of systemic infection after intraperitoneal virus application has shown that MI is based on the expansion of CD8 T-cell clonotypes that differ between individual mice, not by statistical error but by biological variance. Specifically, some clonotypes persisted for the entire observation period in all mice tested individually and longitudinally, although with expanding and contracting clone sizes in the time course. Other clonotypes disappeared or appeared, leading to individual clonotype patterns. Thus, to describe an averaged course of MI, systematic differences in individual responses were leveled out by pooling samples for each time of analysis. Lungs were included in the analysis, because they represent a relevant extra-lymphoid organ site of CMV pathogenesis, where protective CD8 T cells are enriched in infiltrates [48].

Functional CD8 T cells specific for a panel of viral epitopes were quantitated by an ELISpot assay based on their capability to secrete IFN-γ upon stimulation with the respective antigenic peptides (Figure 2A and Figure 3A). The advantage of the ELISpot assay compared to MHC-peptide multimer and intracellular cytokine staining is the fact that it is not biased by gating decisions. The acute response in spleen and lungs, measured at 1 wk, essentially confirmed the known epitope hierarchy for the acute immune response to mCMV in immunocompetent BALB/c mice after intraplantar infection, defining immunodominant and subdominant epitopes [49]. Note that relative cell numbers revealed an enrichment of epitope-specific CD8 T lungs in the lungs compared to the spleen, also after intraplantar infection. In the contraction phase at around 12 wks, frequencies of CD8 T cells had declined for all epitopes, without a notable change in their hierarchy. The contraction was followed by an increase in frequencies, preferentially for the epitopes IE1 and m164, during latent infection. Notably, these are the same two epitopes defined previously as the prototypic MI-driving epitopes in the HCT model [45,50], as well as after high-dose systemic/intravenous infection of immunocompetent mice [51]. So, as far as functional cells are concerned, there exists MI also after intraplantar infection.

**Figure 4:**
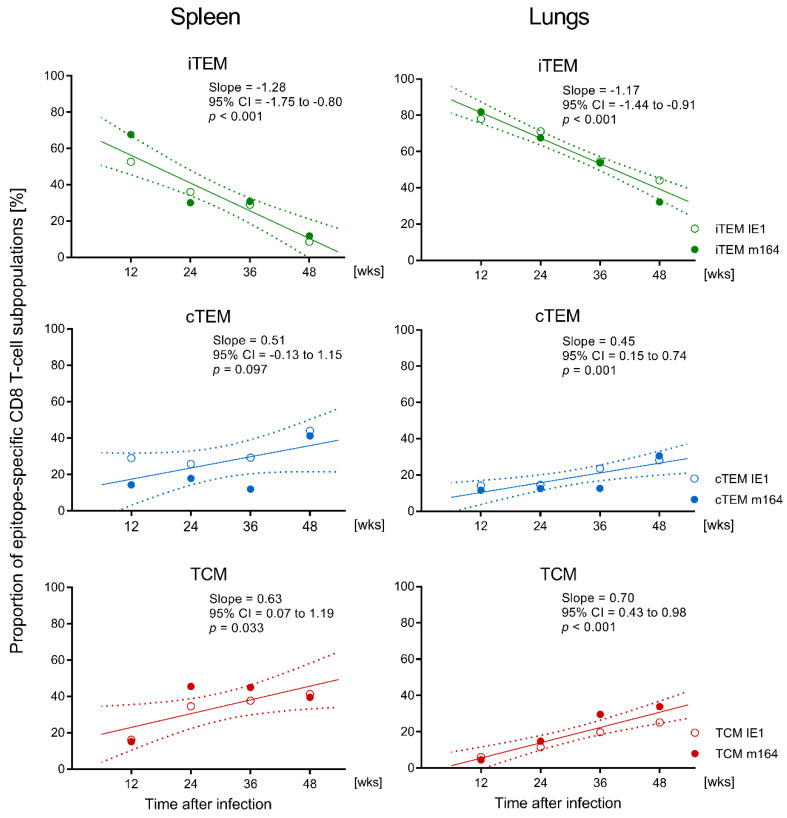
Trend analysis of CD8 T-cell memory population dynamics after intraplantar infection. Data from times during latent infection (wks 12, 24, 36, and 48) shown in Figure 2B (spleen) and Figure 3B (lungs) were subjected to linear regression analysis for determining the statistical significance of loss of iTEM (negative slope) and the corresponding rise of cTEM and TCM (positive slopes) over time. Dotted curves represent the 95% confidence intervals (CI). Trends are considered significantly different to the null hypothesis of no trend (slope 0) for *p* < 0.05.

In parallel to the functional assay, and using the same pools of CD8 T cells, proportions of iTEM, cTEM, and TCM were determined for MHC-peptide multimer-stained cells expressing TCR specific for presented IE1 and m164 peptides (Figure 2B and Figure 3B). For both epitopes, the pool of iTEM in the spleen declined during latent infection between wk 12 and wk 48, and correspondingly, the population became increasingly dominated by cTEM and TCM (Figure 2B). As expected for an extra-lymphoid site, proportions of iTEM were generally higher in the lungs compared to the spleen. However, the general trend of iTEM declining over time, paralleled by increasing proportions of cTEM and TCM, applied also to the epitope-specific populations of pulmonary CD8 T cells (Figure 3B). Trend analysis by linear regression revealed linearity and statistical significance of the decline in iTEM and the corresponding increase in cTEM, and even more in TCM, in both spleen and lungs, after intraplantar infection (Figure 4).

**Figure 5:**
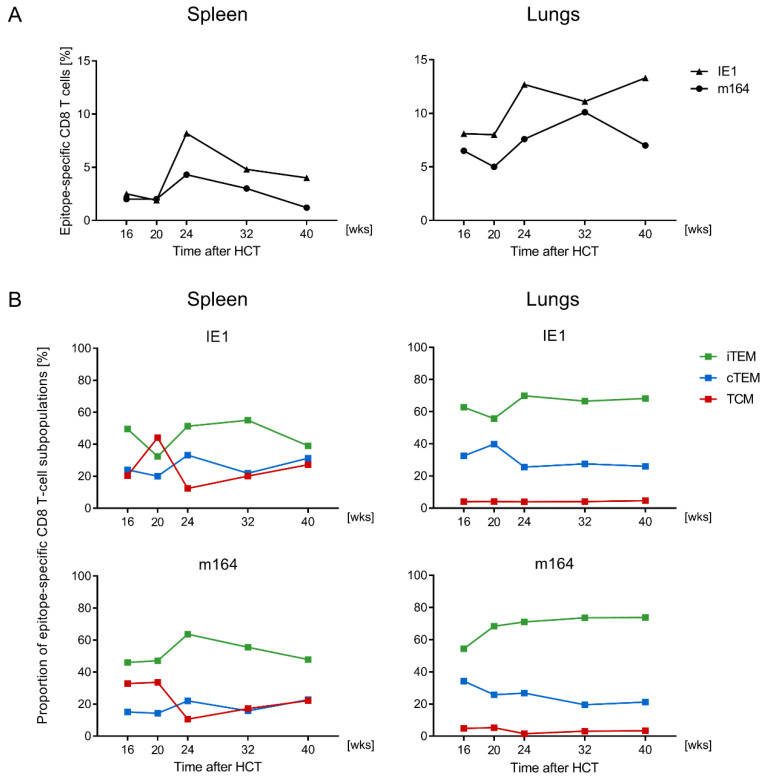
Kinetics of the viral epitope-specific CD8 T-cell response in the HCT model of MI. At the indicated times after HCT and intraplantar infection with 10^5^ PFU of mCMV, CD8 T cells were isolated from spleen and lungs (pool of 5 organs per time of assay) of the HCT recipients. (**A**) Time course of the response to MI-driving epitopes IE1 and m164. (**B**) Corresponding dynamics of memory CD8 T-cell populations iTEM (green symbols), cTEM (blue symbols), and TCM (red symbols).

For a comparison, using the same site of infection and the same virus dose, MI specific for the known MI-driving epitopes IE1 and m164 is dominated by iTEM in spleen and lungs in the HCT model (Figure 5). Note, that in HCT, hematoablative conditioning of the recipients annuls immune control in the draining PLN. This results in systemic infection despite local virus application, so that MI in HCT after intraplantar infection is similar to MI after intraperitoneal or intravenous infection of immunocompetent mice. We have recently documented high dynamics of the CD8 T-cell response after HCT, differing between individual HCTs, despite an identical experimental set-up [50]. Here, we found that IE1-specific MI, and to a lesser extent m164-specific MI, collapsed in the spleen after wk 24, but was maintained in the lungs for a longer period with some difference between the epitopes (Figure 5A). Importantly, in the HCT model, epitope-specific populations in the lungs were dominated throughout the time course by CD62L^−^ populations iTEM > cTEM, whereas CD62L^+^ TCM were found primarily in the spleen, where they increased from wk 24 onward (Figure 5B). This difference in lymphoid and extra-lymphoid localization is consistent with the fact that CD62L, which defines TCM in non-naïve mCMV-specific CD44^+^ CD8 T-cell populations [27], is involved in lymphocyte homing to lymphoid tissues [52,53].

As the key message, this set of experiments has revealed a fundamental difference in the CD8 T-cell population dynamics between local infection and systemic infections. Whereas, in systemic infections, iTEM dominate the response, local infection is associated with a continuous loss of iTEM and corresponding establishment of memory constituted by cTEM and, in particular, by TCM. 

### 3.2. Functional Avidity of CD8 T cells Is Decisive for Overcoming Viral Immune Evasion

**Figure 6:**
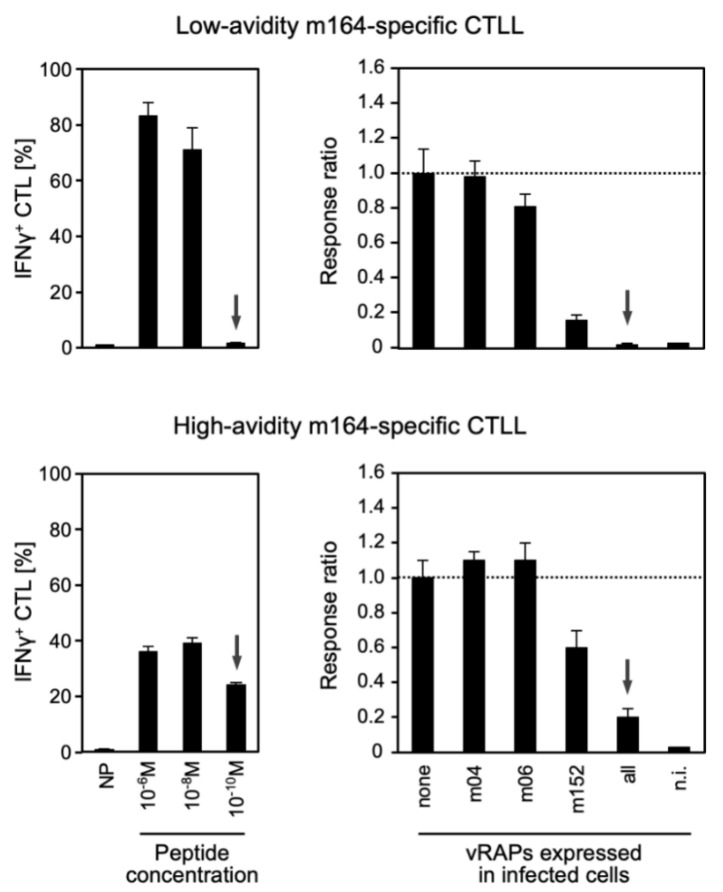
vRAP-modulated recognition of infected cells depending on CD8 T-cell functional avidity. CTLL differing in functional avidities for the D^d^-presented m164 epitope were generated by repetitive stimulation of mCMV-primed memory CD8 T cells with the corresponding synthetic antigenic peptide at concentrations of 10^−8^ M (positively selecting low avidity cells) and 10^−10^ M (positively selecting high avidity cells). (Left panels) Recognition of MEF target cells by the m164 epitope-specific low and high avidity CTLL cells after exogenous loading with synthetic m164 peptide at the indicated molar concentrations. NP, negative control with no peptide loading. Arrows point to the decisive message highlighting the difference between low and high avidity. Bars show the proportion of cells responding in the ELISpot assay by secretion of IFN-γ, error bars indicate the 95% confidence intervals. (Right panels) Corresponding recognition of MEF infected with a panel of recombinant mCMVs selectively expressing the indicated ‘viral regulators of antigen presentation’ (vRAP). None, no vRAP expressed after infection with the triple gene-deletion mutant mCMV-ΔvRAP. All, vRAPs m04, m06, and m152 expressed after infection with the parental virus that corresponds to wild-type mCMV. n.i., uninfected MEF. Frequencies of responding cells were normalized to the response against MEF not expressing vRAPs after infection with the triple gene-deletion mutant. Bars represent response ratios, with ratio 1.0 defined by the most probable number determined for the triple gene-deletion mutant. Error bars represent the 95% confidence intervals. The arrows highlight the impact of functional avidity on the recognition of cells expressing all vRAPs.

Frequencies of functional epitope-specific CD8 T cells are usually determined under saturating stimulation conditions aimed at detecting all cells that express TCR specific for the respective MHC-I-presented antigenic peptides (pMHC-I complexes). However, for polyclonal populations, interaction between TCR and pMHC-I shows a Gaussian avidity-distribution, ranging from low to high avidity. Previous work has revealed that TCR with high structural avidity have a better chance to interact with limited numbers of pMHC-I complexes at the surface of infected cells, which correlates with a higher protective capacity of the T cells [28]. The biologically important functional avidity [26] integrates structural TCR avidity, TCR expression density, and co-receptors that stabilize the interaction between T cells and infected target cells presenting pMHC-I complexes. High avidity interactions are particularly important for the recognition of CMV-infected cells, because hCMV, as well as mCMV, express immune evasion molecules [1,2], also referred to as ‘viral regulators of antigen presentation’ (vRAP) [54]. These proteins interfere with pMHC-I cell surface expression, resulting in limited numbers of pMHC-I complexes available for recognition by T cells. Functional avidity was estimated here by exogenous loading of uninfected cells with graded molar concentrations of antigenic peptides, starting with a saturating concentration of 10^−6^ M. Although exogenous and endogenous peptide loading of MHC-I molecules are not equivalent, one can define the concentration of exogenous synthetic peptides that functionally corresponds to the recognition of infected cells in which the MHC-I molecules become endogenously loaded with the respective naturally-processed peptides (Figure 6).

Whereas cells of a low avidity CTLL specific for the MI-driving m164 epitope recognized MEF exogenously loaded with a peptide concentration of ≥ 10^−8^ M but not with 10^−10^ M, cells of a high avidity CTLL of the same specificity still recognized cells at a peptide loading concentration of 10^−10^ M (Figure 6, left panels). Based on this, we defined 10^−9^ M as the borderline peptide loading concentration, separating low and high avidity. One may wonder why the response rate was low in the high avidity CTLL compared to the low avidity CTLL. An explanation may be that high avidity interaction and the resulting extensive signaling lead to exhaustion preferentially of high avidity cells, as shown recently by Schober and colleagues [55] and discussed below for selective loss of high avidity clones in vivo.

Notably, MEF infected with mCMV expressing all three currently known vRAPs were recognized only by the high avidity CTLL (Figure 6, right panels). It was previously shown that the vRAP m04/gp34, when expressed alone, does not inhibit pMHC-I cell surface expression, whereas vRAPs m06/gp48 and m152/gp40 both have immune evasion function by inhibiting pMHC-I cell surface expression, and thus the recognition of infected cells, with a strength of m152 > m06 [54]. Interestingly, when cells were infected with vRAP gene deletion mutants of mCMV expressing the vRAPs individually, an inhibitory function of m06 was only visible with the low-avidity CTLL, and in accordance with this, the inhibition of target cell recognition by m152 appeared to be stronger when tested with the low avidity line, compared to the high avidity line. These data resolve the misconception that immune evasion molecules would completely prevent cell surface display of pMHC-I, a view that is based on data published for CTLL with undefined, and presumably too low, functional avidities [56]. In addition, assay type and sensitivity are important for detecting limited peptide presentation in the presence of immunoevasive vRAPs. Specifically, cells of a CTLL recognizing IE1 peptide at exogenous loading concentrations of ≤10^−9^ M were stimulated by infected cells for secretion of IFN-γ but failed in the cytolysis assay [54]. Importantly, in vivo protection was found to correlate with IFN-γ secretion rather than with in vitro cytolytic activity [54,57].

As a quintessence of these findings, protective CD8 T cells are expected to require a functional avidity corresponding to an exogenous peptide loading concentration of ≤10^−9^ M in order to recognize infected cells and protect against CMV disease, despite the expression of viral immune evasion genes.

### 3.3. Avidity Dynamics of Functional Viral Epitope-Specific CD8 T cells

**Figure 7:**
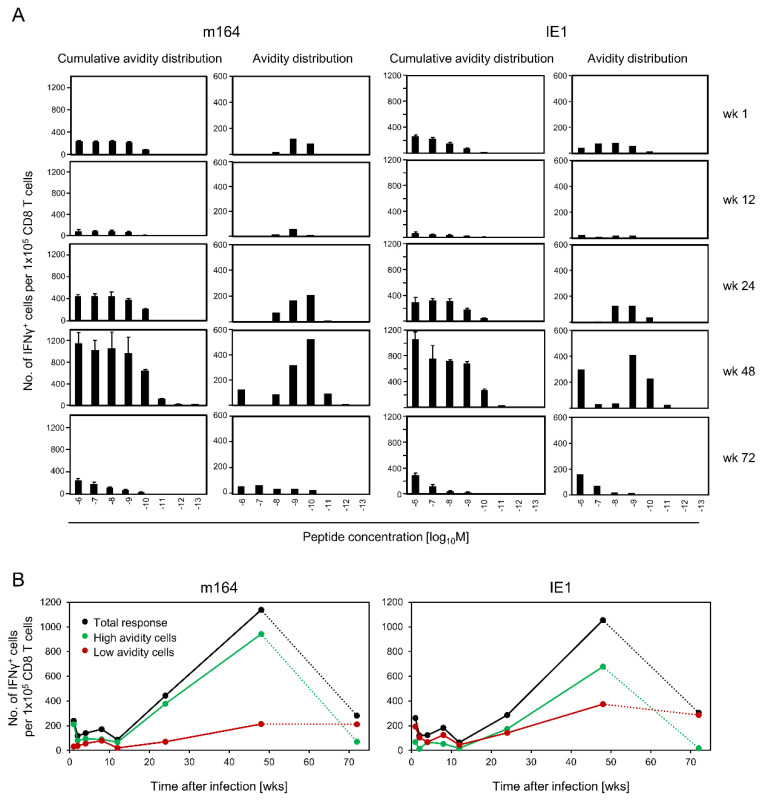
Kinetics of m164- and IE1-epitope-specific functional avidity patterns. (**A**) Immunomagnetically purified CD8 T cells derived from spleens of BALB/c mice (pools of 10) were tested at the indicated times after intraplantar infection with 10^5^ PFU of mCMV. Bars indicate numbers of CD8 T cells responding in the ELISpot assay with IFN-γ secretion to stimulation by P815 cells exogenously loaded with synthetic peptide at the graded molar concentrations indicated. Cumulative avidity distributions reveal frequencies of cells responding to the indicated concentration tested, which includes cells that also respond to lower concentrations. Error bars represent 95% confidence intervals. Gaussian-like avidity distributions reveal frequencies of cells responding exactly to the peptide loading concentration indicated. These are deduced from the cumulative avidity distributions by plotting the difference between neighboring cumulative frequencies. (**B**) Preferential MI of high avidity CD8 T cells in the time course. Data from the avidity distributions shown in **A** were classified into high avidity recognition (green solid and dotted lines: ≤10^−9^ M) and low avidity recognition (red solid and dotted lines: >10^−9^ M). Black lines: sum of cells of all avidities.

With this insight in mind, we revisited MI in the spleen after intraplantar infection of immunocompetent mice in due consideration of functional avidity of CD8 T cells specific for the MI-driving epitopes m164 and IE1 (Figure S2, reproduced in a separate experiment with prolonged observation time in Figure 7). ‘Cumulative avidity distributions’ reveal the numbers of cells responding to target cells exogenously loaded with graded molar concentrations of the respective peptide, which implies that frequencies measured at a defined concentration sum up cells responding to this concentration and all lower concentrations. In contrast, based on the same data, ‘Gaussian avidity distributions’ reveal the number of cells responding to target cells loaded with precisely the indicated peptide concentration. At a glance, after a marked contraction at wk 12, MI occurred and was made up by cells of ‘protective avidity’ (corresponding to ≤10^−9^ M) and of ‘non-protective’ avidity (>10^−9^ M) (Figure 7A). Curiously, while MI for both epitopes essentially represents an increase in the numbers of high avidity cells, a population with very low avidity, corresponding to a peptide loading concentration of 10^−6^ M, expanded in a late phase of ongoing MI measured at wk 48. These results are summarized in graphs, showing numbers of IE1 as well as m164 epitope-specific CD8 T cells in the time course, and classified according to high or low avidity (Figure 7B). Using the total response, which includes all avidities, for comparison, the graphs highlight that MI is based primarily on the expansion of high avidity memory T cells.

Unexpectedly, when the latently infected mice had reached the stage of senescence at the quite late time of wk 72, a secondary contraction phase was observed that is characterized by a preferential loss of high avidity cells (Figure 7B). As the remaining cells are mostly of non-protective avidity, this gives the important information that CMV vector-based vaccines may not confer lifelong immunity.

## 4. Discussion

Our data have revealed two important new findings in the model of intraplantar mCMV infection: (1) MI of viral epitope-specific functional CD8 T cells proved to be based on TCM and cTEM, instead of on iTEM previously found to dominate in models of systemic infections, including the systemic infection of transiently immunocompromised HCT recipients. Whereas one might argue that MI of TCM and cTEM in the model of intraplantar infection is quantitatively far less impressive than MI of iTEM after systemic infection, we believe that our finding of an expanding TCM pool is in fact promising news for promoting CMV as a vaccine vector for use in humans in whom vaccines that depend on systemic infection are impracticable. (2) MI is associated with ‘avidity maturation’ by a selection of epitope-specific CD8 T cells of ‘protective functional avidity’. High avidity is definitively always an advantage in terms of protective capacity, even if the vaccine target should not express equivalents of CMV immune evasion proteins. Many pathogens and basically all tumors also have evolved strategies of immune evasion that limit antigen presentation and call for high avidity recognition. High avidity CD8 T cells have a better chance to become activated and secrete IFN-γ despite very few pMHC-I complexes being displayed at the cell surface of infected cells under conditions of immune evasion. In the case of mCMV, IFN-γ by itself is not a direct antiviral effector molecule [58], but it inhibits virus assembly in combination with TNF-α [59] and it relieves immune evasion by enhancing the presentation of pMHC-I complexes [58,60]. Thus, by enhancing antigen presentation, IFN-γ produced by high avidity cells in the first place can subsequently recruit also low avidity cells for antigen recognition and protection.

Avidity maturation can be explained by limited peptide presentation during viral latency, based on low transcription incidence and low numbers of TEL coding for antigenic peptides. The paucity of pMHC-I cell surface complexes on latently infected non-hematopoietic tissue cells (reviewed in [6,25]) logically requires high avidity interaction for T-cell stimulation and thus favors selection of pre-existing high avidity T-cell clones, with clone sizes increasing over time based on repeated restimulations. In line with this interpretation, recent work led to the conclusion that the inflationary T-cell pool is comprised mainly of high avidity CD8 T cells, outcompeting lower avidity CD8 T cells, and that the amount of early-primed KLRG1^-^ cells and the number of cells with a central memory phenotype are a critical determinant for the overall magnitude of the inflationary T-cell pools [61,62]. Our findings add the information that avidity maturation by positive selection in polyclonal CD8 T-cell populations applies not only to the pool of iTEM, but also to the pools of cTEM and TCM.

In seeming contradiction to avidity maturation during MI, we casually noticed at a late time of still ongoing MI an unpredicted increase in cells with a very low avidity that corresponded to an exogenous peptide loading concentration of 10^−6^ M. The fact that this phenomenon was observed for both MI-driving mCMV peptides makes a cross-reactive ‘molecular mimicry’ recognition by cells specific for an unrelated antigenic peptide based on TCR degeneracy [63,64,65,66] less likely, although one cannot formally exclude an incidental existence and the consequent expansion of T cells of two different unrelated specificities, each cross-recognizing either of the two mCMV epitopes with low avidity. A recurring acute response to a reactivated productive infection might be discussed as an alternative explanation. We consider this also unlikely, as for both epitopes, the avidity distributions during the acute response at wk 1 were clearly different to the patterns seen at wk 48, when only cells recognizing 10^−6^ M peptide had expanded to levels higher than during the acute response. Regardless of the unknown nature of these low avidity cells, they will not mediate functional ‘heterologous immunity’ [66], as based on their exceedingly low avidity, they will not recognize limited mCMV-antigen presentation after endogenous antigen processing.

After an observation time of 72 wks, when mice had reached senescence, a secondary contraction was observed, during which primarily the pool of high avidity cells collapsed. Interestingly, Schober and colleagues [67] discussed a model predicting such a preferential loss of high avidity T-cell clones at late stages following MI, due to ‘proliferative senescence’, and evidence in support of this model has been provided just recently [55]. Our data are consistent with this finding and indicate that loss of high avidity cells is not just the fate of individual clones but indeed applies also to polyclonal populations and to different epitopes. Furthermore, our observations refer to the immunocompetent host in which T-cell clones cannot develop freely but compete for proliferation niches and growth factors, and may be subject to immunoregulatory networks. This is important, as it represents a situation closer to real-life complexity in a healthy human vaccinee than are studies on individual, barmarked clonotypes in an immunodeficient host environment.

The secondary contraction may be discussed to relate to the more recently disputed phenomenon of immunosenescence/immunoexhaustion in the elderly host [68,69,70,71]. It is important to call to mind that the recognition of infected cells, which is the basis for protection, requires functional avidities that correspond to exogenous peptide loading concentrations of ≤10^−9^ M (this report). As the functional avidities of those cells that survived the secondary contraction were mostly lower, corresponding to >10^−9^ M, the loss of high avidity clones predictably results in loss of protective capacity of the CD8 T-cell population. Thus, immunosenescence is not associated with a still ongoing MI, but rather with the collapse of MI and loss of high avidity cells at very late stages. This may explain why CMV-specific immunosenescence is not consistently observed. 

The key question remains why MI, after an intraplantar infection of the immunocompetent host, differs from systemic infection models. A clue to an answer is given by the overall very low latent viral genome load of only 10–100 viral genomes in the lungs that is established following limited virus replication in the acute phase under conditions of efficient immune control (Figure S1). This is the decisive difference to the HCT model where extensive virus replication and spread during acute infection were found to result in a ≈100-fold higher viral genome load in latently infected lungs [72,73], which leads to more frequent events of TEL transcription and TEL-derived peptide presentation that drives the expansion of the iTEM pool [25]. Consistent with such a causal relation, mathematical modeling revealed that the time course of iTEM-based MI is best described by a model that assumes frequent restimulation events [74]. With this fundamental understanding, it is predictable and almost trivial that any modulation of the immune control during acute infection, and thus a modulation of viral load, has an impact on MI during latent infection, as exemplified for immune modulation by IL-10 [75]. Efficient immune control of acute infection results in low load and dampening of iTEM-based MI, whereas inefficient immune control of acute infection results in high load and fueling of MI.

**Figure 8:**
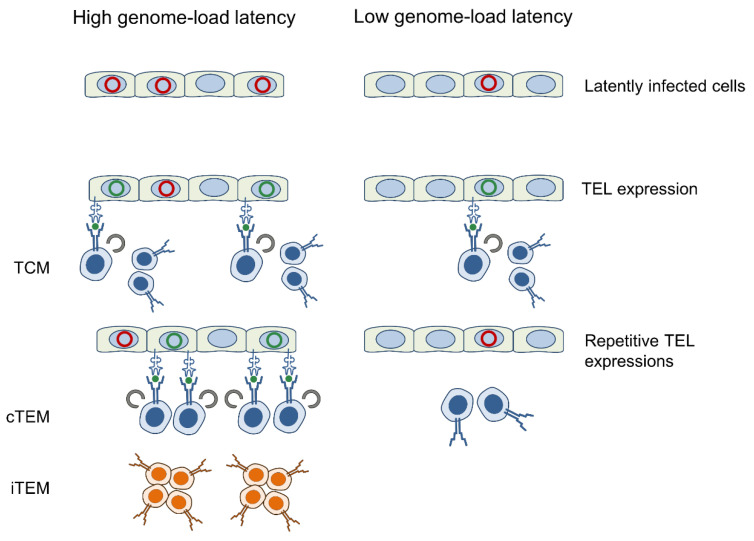
Concluding hypothesis. For detailed explanation, see the section ‘Discussion’. Red circles: Silenced viral genomes in the nucleus of latently infected cells. Green circles: Latent viral genomes transcriptionally desilenced at a gene locus coding for an antigenic peptide. Receptor symbols on latently infected cells and memory CD8 T cells represent peptide-loaded MHC-I molecules and TCR, respectively.

According to an established model of linear memory CD8 T-cell differentiation upon repetitive restimulations leading from TCM to cTEM, and finally to iTEM [76], we propose the hypothesis that high latent viral genome load, associated with high antigen-encoding TEL activity, results in many rounds of T-cell restimulation that drive proliferation and differentiation to the stage of iTEM. In contrast, low latent viral genome load, which is associated with low antigen-encoding TEL activity and accordingly fewer rounds of restimulation of TCM, generates primarily cTEM, but not iTEM (Figure 8).

## 5. Conclusions

MI, when based on the expansion of iTEM, is no longer a valid argument for CMV-based vaccine vectors. We believe, however, that it remains worthwhile to pursue the concept, because the efficient generation of TCM with ‘stemness’ and high-proliferative potential for mounting an efficient recall response [32] is anyway preferable over an increasing level of iTEM that might even have raised safety concerns, due to their permanently activated state also in the absence of the vaccine target pathogen.

## Supplementary Materials

The following are available online at https://www.mdpi.com/2076-393X/8/3/402/s1, Figure S1: Verification of viral latency in spleen and lungs, Figure S2: Kinetics of m164- and IE1-epitope-specific functional avidity patterns.
