# Supplementary material for: Revisiting CD8 T-cell ‘Memory Inflation’: New Insights with Implications for Cytomegaloviruses as Vaccine Vectors
Source: Vaccines (Basel). 2020 Jul 22;8(3):402. doi: 10.3390/vaccines8030402 (PMC7563500; doi:10.3390/vaccines8030402)
Supplement: Supplementary file 1 [file vaccines-08-00402-s001.pdf]

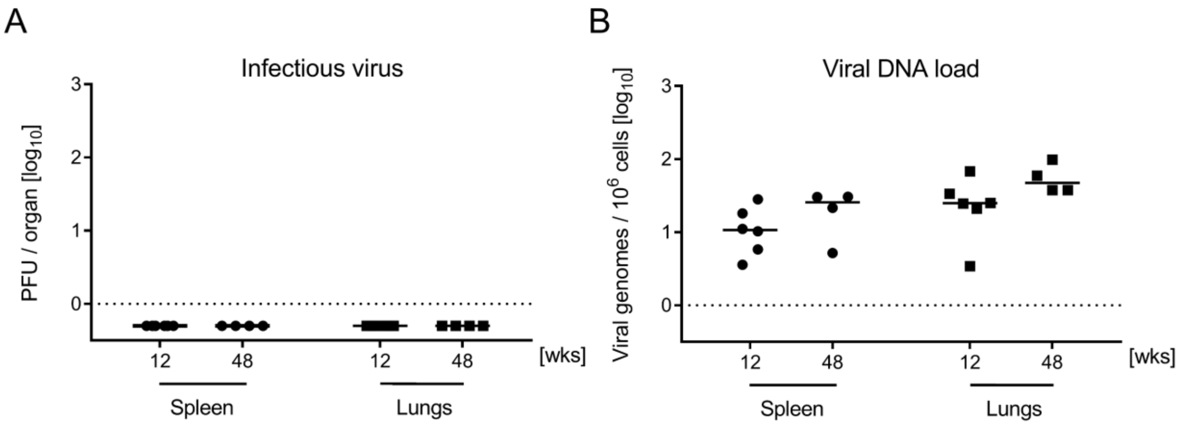

Supplementary Figure S1 Verification of viral latency in spleen and lungs. (**A**) Absence of infectious virus (PFU, plaque-forming units) and (**B**) presence of viral genomes in spleen and lungs at the indicated times after intraplantar infection of immunocompetent BALB/c mice with 10^5 PFU of mCMV. Symbols show data for individual mice with median values indicated by horizontal bars. Dotted lines mark the detection limits of the respective assays.

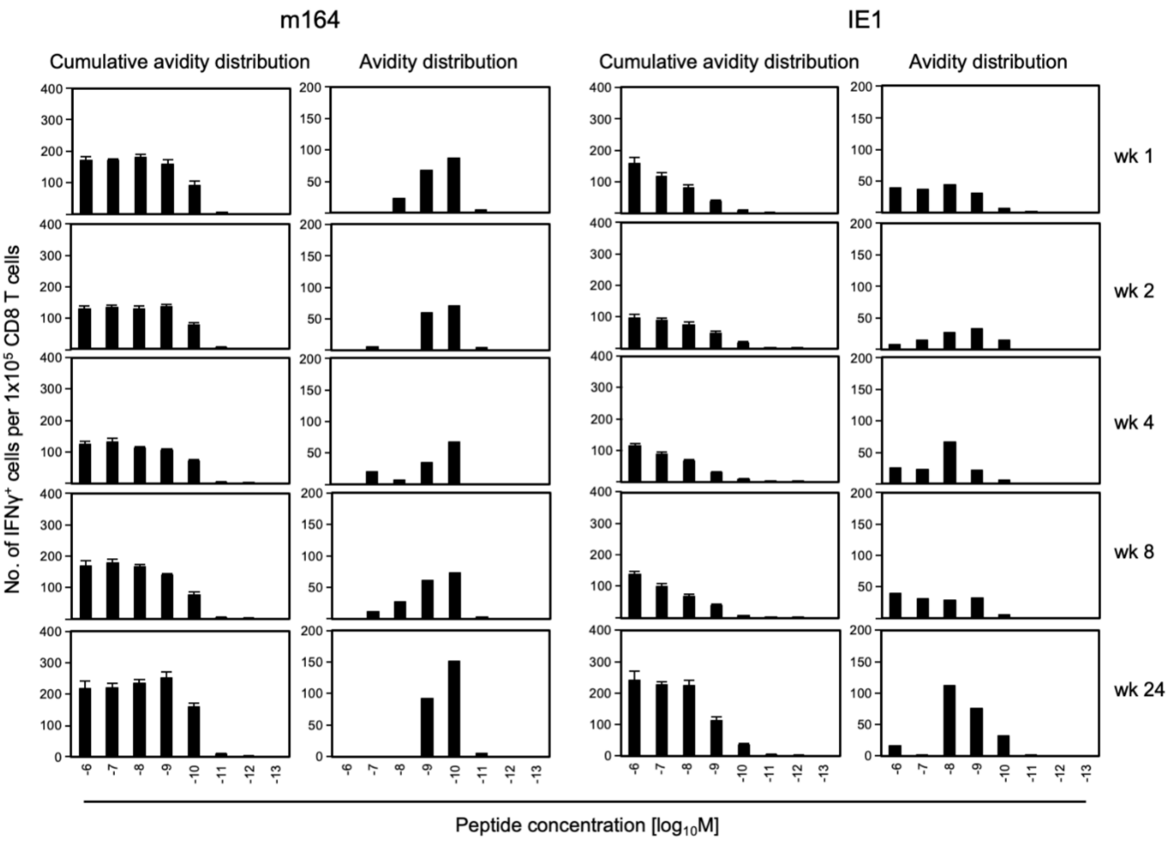

Supplementary Figure S2 Kinetics of m164- and IE1-epitope-specific functional avidity patterns. Immunomagnetically purified CD8 T cells derived from spleens of BALB/c mice (pools of 10) were tested at the indicated times after intraplantar infection with 10^5 PFU of mCMV. Bars indicate numbers of CD8 T cells responding in the ELISpot assay with IFN-γ secretion to stimulation by P815 cells exogenously loaded with synthetic peptide at the graded molar concentrations indicated. Cumulative avidity distributions reveal frequencies of cells responding to the indicated concentration tested, which includes cells that respond also to lower concentrations. Error bars represent 95% confidence intervals. Gaussian-like avidity distributions reveal frequencies of cells responding exactly to the peptide loading concentration indicated. These are deduced from the cumulative avidity distributions by plotting the difference between neighboring cumulative frequencies.
